# Outcomes of switching from protease inhibitor-based antiretroviral therapy to bictegravir/emtricitabine/tenofovir alafenamide (B/F/TAF) in virologically suppressed adults with nucleos(t)ide analogue resistance– a phase IV randomised, open-label study (PIBIK study)

**DOI:** 10.1186/s12985-025-02648-3

**Published:** 2025-02-10

**Authors:** Collins Iwuji, Laura Waters, Ana Milinkovic, Chloe Orkin, Julie Fox, Frank Post, Nicky Perry, Chloe Bruce, Natalie Dailey, Ye To, Stephen Bremner, Duncan Churchill, Anna Maria Geretti

**Affiliations:** 1 Department of Global Health and Infection, Brighton and Sussex Medical School, University of Sussex, Falmer, Brighton, UK; 2 Africa Health Research Institute, KwaZulu-Natal, South Africa; 3 The Mortimer Market Centre, Central and Northwest London NHS Foundation Trust, London, UK; 4 Chelsea and Westminster Hospital NHS Foundation Trust, London, UK; 5 Blizard Institute, Faculty of Medicine and Dentistry, Queen Mary University of London, London, UK; 6 Barts Health NHS Trust, London, UK; 7 Guy’s and St Thomas’ NHS Foundation Trust, London, UK; 8 King’s College Hospital NHS Foundation Trust, London, UK; 9 University Hospitals Sussex NHS Foundation Trust, London, UK; 10 Brighton & Sussex Clinical Trials Unit, University of Sussex, Brighton, UK; 11 Department of Primary Care and Public Health, Brighton and Sussex Medical School, University of Sussex, Brighton, UK; 12 Department of Systems Medicine, University of Rome Tor Vergata, Rome, Italy; 13 Royal Free London NHS Foundation Trust - North Mid, London, UK; 14 School of Immunity & Microbial Sciences, King’s College London, London, UK

**Keywords:** HIV, Drug resistance, Archive, Bictegravir, Tenofovir alafenamide, Integrase strand transfer inhibitor, Boosted protease inhibitor, Switch

## Abstract

**Background:**

There are limited data on how historical nucleoside reverse transcriptase inhibitor (NRTI) resistance-associated mutations (RAMs) other than M184V/I, affect the activity of B/F/TAF. We evaluated the outcomes of switching virologically suppressed (HIV-1 RNA < 50 copies/mL) individuals harbouring major RAMs from boosted protease inhibitor (bPI)-based therapy to B/F/TAF.

**Methods:**

Participants had various historical genotypic patterns including M184V/I, ≤2 thymidine analogue mutations (TAMs), and other NRTI RAMs (NAMs), and no integrase resistance. Baseline RAMs were explored by retrospective sequencing of cellular HIV-1 DNA. Participants were randomised (1:1) to switching to B/F/TAF either immediately or after 24 weeks. The primary outcome was the proportion of participants maintaining virological suppression (pure virologic response) at week-24; secondary outcomes were proportion of participants with virological suppression at week-48, pre-specified safety measures, and treatment-emergent resistance.

**Results:**

Historically, 21/72 (29.2%) participants had M184V/I, 5 (6.9%) M184V/I + 1 NAM, 31 (43.1%) 1 TAM ± M184V/I ± 1 NAM, and 15 (20.8%) 2 TAMs ± M184V/I ± 1 NAM. At week-24, proportions maintaining virological suppression were 33/33 (100%) on B/F/TAF vs. 38/39 (97.4%) on bPI (difference 2.6%; 95% CI -2.4%, 7.5%). Drug-related adverse events (AEs) were reported in 10/33 (30.3%) vs. 1/39 (2.6%), respectively. The immediate switch arm had improved lipid parameters but increased HbA1c and weight. Virological suppression was maintained at week-48. There were six discontinuations; four on B/F/TAF were drug-related and the two on bPI were not drug-related.

**Conclusions:**

Historical NRTI resistance did not compromise the effectiveness of B/F/TAF in virologically suppressed adults. 12% experienced treatment-limiting AEs after switching.

**Registration:**

EudraCT no: 2018-004732-30

## Background

Regimens comprising a boosted protease inhibitor (bPI) plus 2 nucleos(t)ide reverse transcriptase inhibitors (NRTIs) have been shown to retain efficacy in the presence of common forms of transmitted or acquired NRTI resistance [1, 2]. The data for integrase strand-transfer inhibitors (INSTIs) are multifaceted. In the SWITCHMRK study, virologically suppressed individuals who switched from a bPI to the first-generation INSTI raltegravir in combination with ≥2 NRTIs showed an increased risk of virological failure, which a post-hoc analysis related to the presence of historical NRTI resistance [3].

Switching from virologically suppressive bPI-based antiretroviral therapy (ART) to regimens based on bictegravir [4] or dolutegravir [5] with 2 NRTIs has been shown to be safe and efficacious. Second-generation INSTIs have greater resilience against resistance relative to first-generation compounds. Clinical trial data show that dolutegravir with 2 NRTIs retains activity in treatment-experienced individuals with NRTI resistance [6, 7]. Nonetheless, data from a large observational cohort indicate that, in individuals receiving dolutegravir, the presence of NRTI resistance increases the risk of treatment-emergent INSTI resistance [8].

Clinical data on the impact of NRTI resistance on the activity of bictegravir/emtricitabine/tenofovir alafenamide (B/F/TAF) fixed dose combination are more limited. Two trials evaluated virologically suppressed individuals switching to B/F/TAF from regimens consisting of either a bPI (atazanavir or darunavir) plus tenofovir disoproxil fumarate/emtricitabine (TDF/FTC) or abacavir/lamivudine (ABC/3TC) (study 1878) [4] or dolutegravir plus ABC/3TC (study 1844) [9]. Both trials demonstrated that switching to B/F/TAF was non-inferior to continuing the baseline regimens over 48 weeks [4, 9]. Documented resistance to any of the study drugs or evidence of previous virological failure were exclusion criteria if identified prior to randomisation [10]. A retrospective analysis of historical genotypic resistance data and of genotypes obtained from cellular HIV-1 DNA of samples drawn at the trial baseline visit identified major NRTI resistance-associated mutations (RAMs) in 89/543 (16.4%) participants in the B/F/TAF arm; these included mainly the 3TC/FTC mutations M184V and M184I, as well as some thymidine analogue mutations (TAMs) [10]. Overall, 86/89 (96.6%) maintained virological suppression at week 48. Based on these retrospective findings, we designed a trial to prospectively investigate the safety and efficacy of switching from a bPI-based regimen to B/F/TAF in virologically suppressed individuals with a historical record of pre-defined patterns of NRTI resistance receiving care in a high-income setting. Historical resistance data were complemented with retrospective genotyping of cellular HIV-1 DNA using peripheral blood mononuclear cells (PBMC) collected at study entry.

## Methods

### Study design and participants

The PIBIK trial was an investigator-initiated phase IV, prospective, multicentre, open label, randomised two arm pilot study to assess the safety and efficacy of switching from a bPI-based regimen to B/F/TAF in virologically suppressed people with HIV who had pre-specified patterns of historical genotypic NRTI resistance. Participants were recruited from seven centres in England, United Kingdom. Eligible participants were adults (18 years and above) on a bPI-based ART regimen with documented plasma HIV-1 RNA < 50 copies/mL for at least 6 months on the current regimen and confirmed at screening. Initially, the protocol specified that participants must not have received INSTIs, but this was later modified to include participants with previous INSTI exposure provided there was no documented virological failure on an INSTI-containing regimen and no documented INSTI resistance. Historical genotypic resistance data were retrieved from each participant; multiple test results were summarised into a cumulative genotype for each individual. Eligible participants had cumulative historical genotypes indicating the presence of NRTI RAMs comprising M184V/I and/or $\:\le\:$2 TAMs (M41L, D67N, K70R, L210W, T215F/Y, K219Q/E/N), and/or other major NRTI RAMs (described as NAMs, e.g., L74I/V, K70E/G/Q), but excluding K65R/N/E, T69ins, and Q151M (with or without A62V, V75I, F77L, F116Y). Presence of NNRTI RAMs was allowed.

We included people of reproductive potential if they were not pregnant or lactating and were using appropriate contraception. The full inclusion and exclusion criteria are described in the study protocol [11]. We obtained written informed consent from each participant before initiation of study procedures.

### Randomisation and masking

The web-based Sealed Envelope™ system was used to allocate individuals randomly to either continue their bPI-based regimen (delayed switch arm) or immediate switch to B/F/TAF (immediate switch arm). The randomisation list was provided by the study statistician and each study site was provided with a randomisation guide.

Participants were stratified based on the three factors resulting in 8 randomisation strata. The stratification factors were:

- The bPI used in the baseline regimen (Atazanavir or Darunavir).
- Number of NRTI RAMs (< 2 vs. ≥2).
- Use of lipid lowering therapy at study day 1 (yes/no).

### Procedures

Participants either continued their bPI regimen (delayed switch arm) or switched to Biktarvy^®^ (immediate switch arm) comprising bictegravir sodium equivalent to 50 mg of bictegravir, 200 mg of emtricitabine, and tenofovir alafenamide fumarate equivalent to 25 mg of tenofovir alafenamide (B/F/TAF). Participants in the immediate switch arm were followed for 48 weeks. In the delayed switch arm, after 24 weeks, participants switched to B/F/TAF and were followed up for a further 24 weeks. The study included a screening period of up to 30 days. Study visits for all participants were planned at baseline and at weeks 4, 12, 24, 28, 36 and 48.

We assessed concomitant medications, adverse events (AEs), and symptom-directed physical examinations at all study visits. AEs were documented using MedDRA (version 21.0) and graded according to the Division of AIDS Grading Scale (version 1.0). Blood tests for haematology, clinical chemistry and plasma HIV-1 RNA load were done at all study visits. Fasting lipids, HbA1c and glucose were done at baseline and at weeks 24 and 48. Weight, body mass index (BMI) and waist circumference were measured at all routine study visits.

Virological failure was defined as a rebound in plasma HIV-1 RNA ≥ 50 copies/mL confirmed at the following scheduled or unscheduled visit, 2 to 3 weeks after the date of the first measured rebound. In cases of confirmed virological failure, eligibility for resistance testing of plasma HIV-1 RNA was a confirmed viral load ≥ 200 copies/mL. The protocol indicated that unless emergent resistance was detected, participants with viral load rebound could remain in the study. Participants could discontinue at the investigator’s discretion or per local treatment guidelines.

Blood samples were taken at baseline for sequencing of cell-associated HIV-1 DNA in isolated PBMC. Testing was conducted retrospectively at the end of the study. The *pol* gene regions encoding the first 99 amino acids of protease, the first 260 amino acids of reverse transcriptase and the first 288 amino acids of integrase were amplified in the diagnostic laboratory of St. Mary’s Hospital in London, using next-generation sequencing (NGS) on the Illumina MiSeq platform. According to the local protocol, results were reported applying a conservative frequency threshold of 15%. Levels of predicted resistance to TAF and FTC were determined using the Stanford Database algorithm (https://hivdb.stanford.edu/). The resistance levels were summarised as follows: None (Stanford susceptible), Low (potential low-level resistance or low-level resistance), Intermediate (intermediate-level resistance) and High (high-level resistance). In the description of levels of resistance, we considered the cumulative historical genotype plus any additional NRTI RAM found in HIV-1 DNA at baseline.

### Outcomes

The primary endpoint was the proportion of participants with HIV-1 RNA < 50 copies/mL at week-24 using pure virological response (PVR_24_).

PVR_24_ was defined as follows [12]:

- On study treatment.
- Absence of confirmed virological rebound, defined as:
  - HIV-1 RNA ≥ 50 copies/mL in 2 consecutive measurements.
  - Single HIV-1 RNA ≥ 50 copies/mL followed by premature discontinuation.
- Individuals who discontinued prior to week-24 for reasons other than virological rebound and with last HIV-1 RNA measurement < 50 copies/mL (i.e., with no HIV-1 RNA data in window) were considered as meeting PVR_24_.

The following secondary outcome measures were planned:

- Change from baseline in serum lipid concentrations, HbA1c, body mass index (BMI), weight, estimated glomerular filtration rate (eGFR), and waist circumference at week 24.
- Proportion of patients with HIV-1 RNA < 50 copies/mL at week-48 (PVR_48_).
- Safety and tolerability of B/F/TAF over 48 weeks.
- Proportion of patients with HIV-1 RNA < 50 copies/mL at weeks 24 and 48 using PVR in those with any archived resistance detected in cellular HIV-1 DNA.
- Emergence of new RAMs in plasma of participants with two consecutive HIV-1 RNA values ≥ 200 copies/mL measured 2–3 weeks apart.

### Sample size

We aimed to recruit 100 participants (50 per arm) into the trial. If there was truly no difference between bPI and B/F/TAF, assuming 90% virological suppression in both arms and 80% power, 98 participants were required to ensure that the upper limit of a two-sided 95% confidence interval (CI) excluded a difference in favour of bPI of more than 17% (the limit of non-inferiority) [13].

### Statistical analysis

The flow of patients through the trial is shown on a flow diagram according to the CONSORT 2010 Statement extension for non-inferiority trials [14]. All randomised patients who received at least one dose of the study medication were included in both the efficacy and safety analysis. Summary statistics were presented by trial arm using median and interquartile range for continuous variables with skewed distributions or mean and standard deviation for normally distributed variables. Categorical variables were summarised using frequencies and percentages. The difference between arms in the proportion with PVR was estimated together with the 95% confidence interval (CI). Missing data were quantified but not imputed. All data were analysed using Stata version 18 (StataCorp. 2023. *Stata Statistical Software: Release 18. College Station*,* TX: StataCorp LLC*.)

## Results

**Fig. 1:**
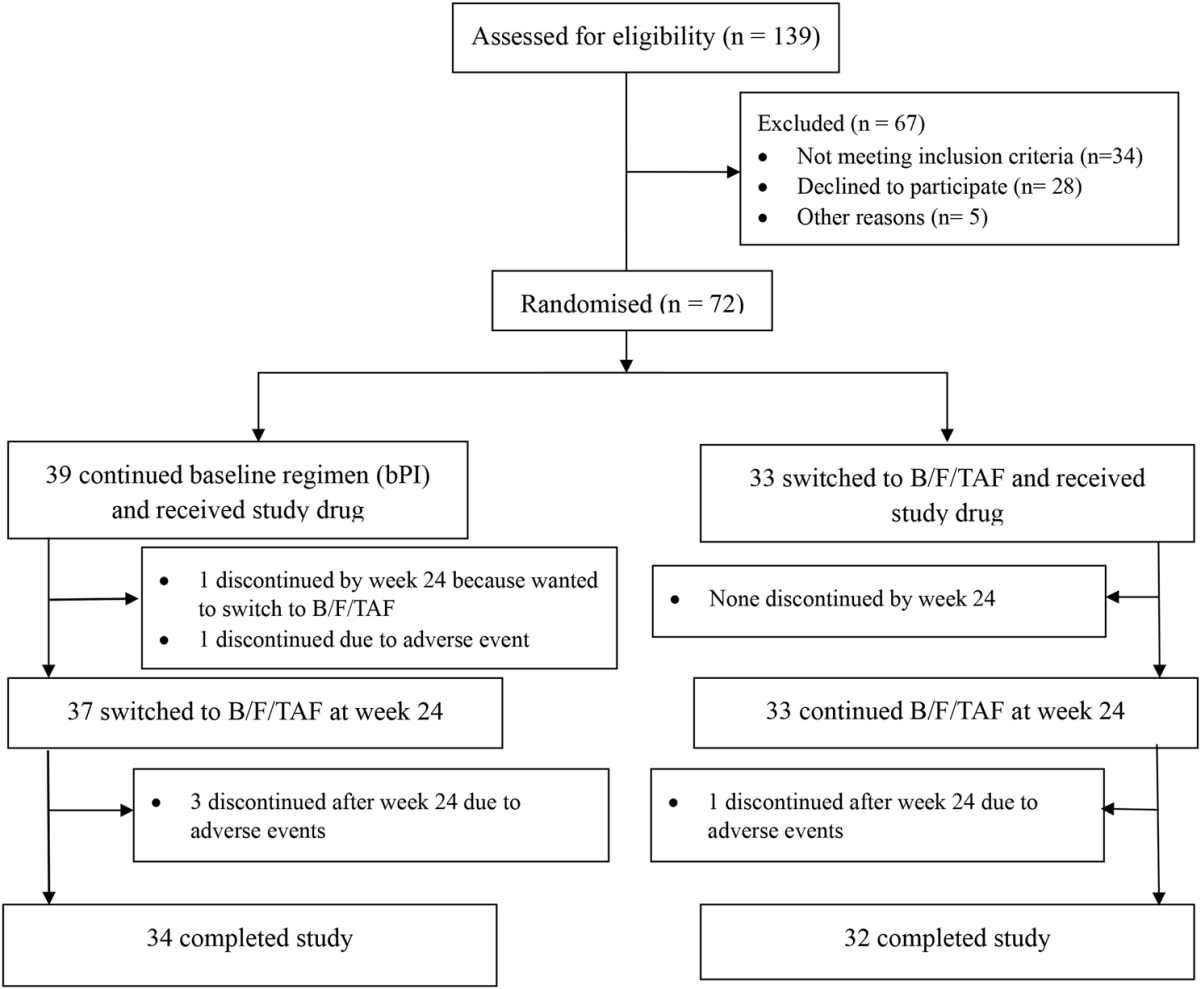
Trial profile

A total of 139 individuals were assessed for eligibility between 16 Sept 2019 and 28 February 2022. Of these, 33 were randomised to immediate switch and 39 to delayed switch (Fig. 1). All received at least one dose of the study drug and were included in the efficacy and safety analyses. The last study visit was on 3 March 2023. Six participants discontinued the study by week 48: four on B/F/TAF due to drug-related adverse events, and two on bPI; one due to a non-related adverse event and the other due to protocol deviation (participant wished to switch to B/F/TAF). All participants had HIV-1RNA < 50 copies/mL at the time of discontinuation.

Demographic and baseline clinical characteristics were well balanced between the treatment groups (Table 1). The study population comprised largely men (64, 88.9%) of White ethnicity (53, 73.6%) with a mean age of 55 years. The median CD4 + T-cell count at baseline was higher in the delayed-switch arm than the immediate-switch arm (632 cells/mm^3^ vs. 560 cells/mm^3^).

**Table 1:** Baseline demographic and clinical characteristics by arm

		Immediate switch arm *N* = 33	Delayed switch arm *N* = 39
Age, mean years (SD)		53 (8)	56 (7)
Self-reported male sex, n (%)		29 (88.0)	35 (90.0)
White race, n (%)		23 (69.7)	30 (76.9)
CD4 T count, median cells/mm^3^ (IQR)^a^		560 (457–800)^a^	632 (453–854)
ART duration, median years (IQR)		30; 17.1 (8.7–21.3)^b^	39; 17.6 (10.9–23.8)
bPI at randomisation, n (%)	Darunavir	28 (84.8)	30 (76.9)
	Atazanavir	5 (15.2)	9 (23.1)
NRTI backbone, n (%)	TDF-based	17 (51.5)	20 (51.3)
	TAF-based	6 (18.2)	7 (17.9)
	Non TDF/TAF-based	4 (12.1)	8 (20.5)
	3TC or FTC	22 (66.7)	31 (79.5)
On lipid lowering drugs, n (%)		11 (33.3)	14 (35.9)
NRTI RAMs in historical genotype^c^, n (%)			
M184V/I alone		12 (36.4)	9 (23.1)
1 TAM		6 (18.2)	10 (25.6)
1 TAM + M184V/I		4 (12.1)	7 (18.0)
2 TAMs		1 (3.0)	3 (7.7)
2 TAMs + M184V/I		2 (6.1)	5 (12.8)
1 NAM + M184V/I		3 (9.1)	2 (5.1)
1 TAM + M184V/I + 1 NAM		1 (3.0)	2 (5.1)
2 TAMs + M184V/I + 1 NAM		3 (9.1)	1 (2.6)
1 TAM + 1 NAM		1 (3.0)	0 (0.0)
NRTI RAMs in baseline genotype^d^			
Any		13 (39.4)	20 (51.3)
None		19 (57.6)	16 (41.0)
Not available		1 (3.0)	3 (7.7)

^a^*n*= 32; ^b^*n*= 30; ^c^Cumulative of all available historical plasma genotypes. ^d^Tested retrospectively using HIV-1 DNA from peripheral blood mononuclear cells collected at study entry. ART = Antiretroviral therapy; PI/b = Boosted protease inhibitor; NRTI = Nucleos(t)ide reverse transcriptase inhibitor; TDF = Tenofovir disoproxil fumarate; TAF = Tenofovir alafenamide; 3TC = Lamivudine; FTC = Emtricitabine; RAMs = Resistance-associated mutations; TAMs = Thymidine analogue mutations; NAM = NRTI RAM other than M184V/I or TAMs

### Historical and baseline resistance

The resistance patterns reported in historical plasma HIV-1 RNA genotypes and those obtained at baseline with cellular HIV-1 DNA are summarised in Table 2.

**Table 2:** Participant-level historical and baseline resistance data with levels of predicted resistance to TAF and FTC

Participant ID	Arm	Historical RAMs^a^	Baseline RAMs^b^	Resistance^c^	
				TAF	FTC
20, 24, 25, 40, 59, 60, 61, 64, 67, 68	IS	M184V/I	None	No	High
45, 47, 55, 62, 70^d^	DS	M184V/I	None	No	High
14	DS	M184V/I	NA	No	High
17	IS	M184V/I	NA	No	High
*21* ^*e*^	DS	M184V/I	M184V	No	High
48	DS	M184V/I	M184V M184I	No	High
30	DS	M184V/I	K219N	No	High
54^d^	DS	M184V/I	M41L D67N K70R M184V T215Y	Interm.	High
27	IS	M41L	None	No	No
28	DS	M41L	None	No	No
11, 36	DS	M41L	M41L	No	No
35, 39	IS	M41L	M41L	No	No
10	DS	M41L	M41L M184V T215Y T125N T215S	Low	High
71	IS	D67N	None	No	No
37^d^	DS	D67N	D67N K219Q	Low	No
16^d^	DS	L210W	K70R	Low	No
6^d^	DS	T215Y	None	Low	No
56	DS	T215F	M184V	No	High
9	DS	K219Q	None	No	No
12	IS	K219N	None	No	No
43	IS	K219N	K219N	No	No
49	DS	K219E	K219E	No	No
2	IS	D67N M184V/I	M184V	No	High
65	IS	D67N M184V/I	D67N M184V	No	High
13, 15	DS	K70R M184V/I	None	No	High
23	IS	K70R M184V/I	None	No	High
26	DS	K70R M184V/I	M184V	No	High
31	DS	M184V/I L210W	NA	No	High
38	IS	M184V/I T215Y	None	No	High
58	DS	M184V/I T215Y	M184V T215Y	No	High
8, 69	DS	M184V/I K219E	None	No	High
46	DS	M41L L210W	NA	Low	No
42^d^	IS	M41L T215Y	M41L M184V M184I T215Y T215D	Low	High
66	DS	D67N K70R	None	Low	No
51	DS	D67N K219Q	D67N K219Q	Low	No
7	DS	D67N K70R M184V/I	None	No	High
19, 53	IS	M41L M184V/I T215Y	None	Low	High
63	DS	M41L M184V/I T215Y	M184V	Low	High
33	DS	M41L M184V/I T215F	M184V T215F T215Y T215I T215N T215S	Low	High
22	DS	K70R M184V/I K219E	None	No	High
72	DS	D67N K70R M184V/I	D67N	No	High
5	DS	K70E M184V/I	M184V	Low	High
32	IS	L74V/I M184V/I	M184I^f^	No	High
29	DS	L74V/I M184V/I	L74V M184V	No	High
34	IS	L74V/I M184V/I	K65R	Interm.	High
18	IS	L74V/I M184V/I	K65R L74V M184V	Interm.	High
50	DS	M41L L74V/I M184V/I	M41L	No	High
52	IS	D67N K70E M184V/I	D67N K70E M184V	No	High
4	DS	L74V/I M184V/I K219E	None	No	High
1	DS	D67N L74V/I M184V/I T215Y	None	Low	High
44	IS	D67N L74V/I M184V/I T215Y	K70Q T215Y T215C T215N T215S	Interm.	High
3	IS	M41L L74V/I M184V/I T215Y	M41L M184V T215Y T215T T215N T215S	Low	High
41	IS	M41L L74V/I M184V/I T215Y	M41L L74I M184V M184I T215Y T215N T215S	Low	High
57	IS	K70E K70R	None	Low	Low

^a^Cumulative historical plasma genotypes obtained by considering all available historical resistance data; ^b^Tested retrospectively by sequencing HIV-1 DNA from peripheral blood mononuclear cells collected at study entry; ^c^Cumulative predicted resistance (Stanford Database algorithm v. 9.6) considering all historical NRTI RAMs combined with any additional NRTI RAM detected at baseline. ^d^Participants who discontinued the study prior to week 48 with HIV-1 RNA < 50 copies/mL; ^e^Participant with confirmed virological rebound. ^f^Occurring in the context of hypermutation. Grey shadowing indicates fully concordant historical and baseline genotypic resistance patterns. DS = Delayed switch arm; IS = Immediate switch arm

When comparing historical and baseline HIV-1 DNA resistance results in 68 participants with baseline resistance data, most (35/68, 51.5%) lacked detectable NRTI RAMs in HIV-1 DNA (Table 2). When RAMs were detected in HIV-1 DNA, 13/68 (19.1%) participants had fully concordant patterns; 10/68 (14.7%) showed major NRTI RAMs in baseline samples that had not been reported historically, including three samples with the tenofovir RAMs K65R and K70Q; and 10/68 (14.7%) showed fewer baseline RAMs.

### Efficacy

Based on the definition of PVR_24_ specified above, all 72 participants that had taken at least one dose of the study drug were included in estimating efficacy. At week-24 (PVR_24_, primary endpoint), 33/33 (100%) in the immediate switch arm vs. 38/39 (97.4%) in the delayed switch arm maintained virological suppression (HIV-1 RNA < 50 copies/mL) [difference in proportions B/F/TAF vs. bPI; 2.6%, (95% CI: -2.4%, 7.5%)]. Week-48 efficacy remained unchanged in terms of proportions with virological suppression in both arms (secondary endpoint).

One participant, who was in the delayed switch arm (ID 21 in Table 2), showed confirmed virological rebound, with HIV-1 RNA levels of 68 copies/mL in the 24 weeks window and 89 copies/mL 3 weeks later. Based on the protocol, the individual remained in the study, switching to B/F/TAF at week 24. At week 48, HIV-1 RNA levels were 77 copies/mL. Testing for emergent resistance was not performed because the viral load never increased ≥ 200 copies/mL. All six participants who discontinued early had a last HIV-1 RNA measurement < 50 copies/mL. When excluding these early discontinuations, overall 65/72 (90.3%) completed 48 weeks with HIV-1 RNA < 50 copies/mL.

### Safety and tolerability

During the first 24 weeks from baseline, AEs were reported in 25 (75.8%) participants in the immediate switch arm and 24 (61.5%) in the delayed switch arm (Table 3). AEs were considered drug-related in 10/33 (30.3%) and 1/39 (2.6%) participants, respectively. Amongst participants in the delayed switch arm, the proportion reporting AEs after switching to B/F/TAF was 14/39 (35.9%) and therefore similar to that observed in the first 24 weeks of the immediate switch arm. Six individuals discontinued study drug: one was due to a protocol deviation in the delayed switch arm and the remaining five were due to adverse events. Of these five, one discontinued at 24 weeks in the delayed switch arm due to a serious adverse event (anal squamous cell carcinoma) and four discontinued on B/F/TAF due to drug related adverse events; two were attributed to weight gain, one to worsening of depression, and one to hypersensitivity.

**Table 3:** Frequencies and percentages of adverse events (AEs) and laboratory abnormalities

	Immediate switch B/F/TAF BL-W24 *N* = 33 (%)	Delayed switch bPI BL-W24 *N* = 39 (%)	Delayed switch B/F/TAF W24-W48 *N* = 39 (%)	Immediate switch B/F/TAF W24-W48 *N* = 33
Any AE	25 (75.8)	24 (61.5)	28 (71.8)	17 (51.5)
Drug related AE	10 (30.3)	1 (2.6)	14 (35.9)	3 (9.1)
Serious AE	0 (0.0)	1 (2.6)	0 (0.0)	0 (0.0)
Discontinuation due to AE	0	1 (2.6)^$^	3 (7.7)^#^	1* (3.0)
AE in ≥ 5% of participants				
• Headaches	4 (12.0)	2 (5.1)	4 (10.3)	
• Hypertension	4 (12.0)			
• Anxiety	3 (9.1)		2 (5.1)	
• Low mood	2 (6.1)		2 (5.1)	
• Abnormal dreams	3 (9.1)			
• Insomnia	3 (9.1)		2 (5.1)	
• Sleep disturbance	3 (9.1)			
• Weight gain	3 (9.1)		2 (5.1)	2 (6.1)
• Tiredness/fatigue	3 (9.1)		3 (7.7)	
• Non-diabetic hyperglycaemia		3 (7.7)		2 (5.1)
Grade 3 or 4 laboratory abnormalities				
• Amylase	1 (3.0)	0 (0.0)	0 (0.0)	0 (0.0)
• Bilirubin	2 (6.1)	1 (2.6)	0 (0.0)	0 (0.0)
• Total cholesterol	0 (0.0)	1 (2.6)	0 (0.0)	0 (0.0)
• LDL cholesterol	0 (0.0)	1 (2.6)	2 (5.1)	0 (0.0)

*Discontinued due to weight gain; $Discontinued due to squamous cell carcinoma of the anus; #Three discontinuations for (i) worsening depression, (ii) weight gain and (iii) hypersensitivity to B/F/TAF

**Fig. 2:**
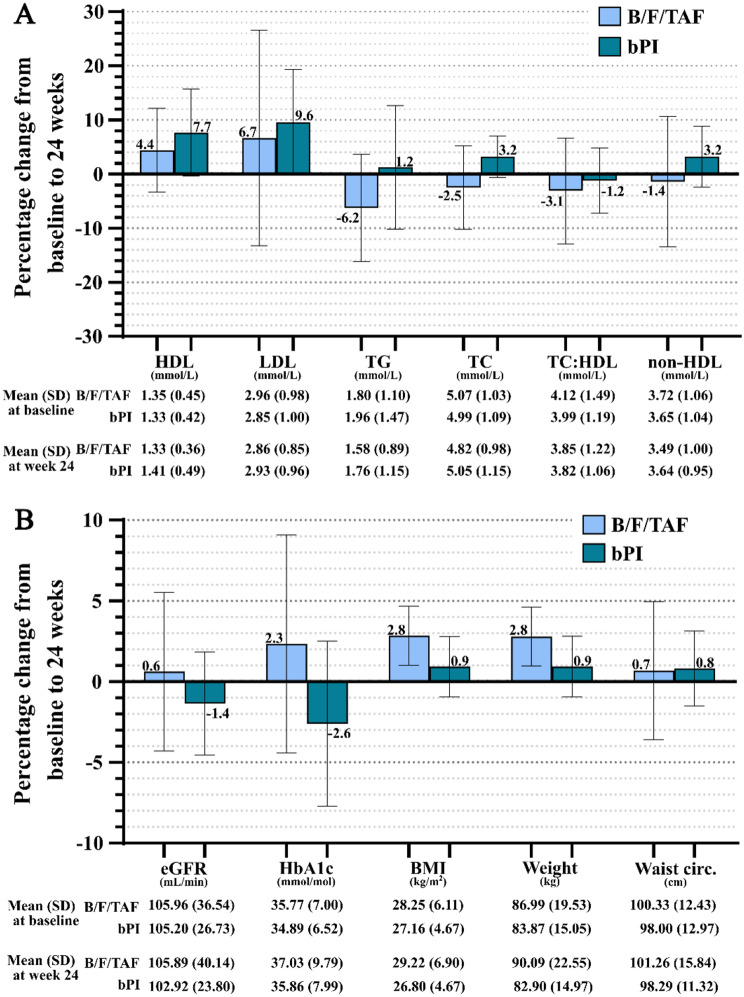
**a**. Percentage change in lipid parameters from baseline to week 24, by arm. **b**. Percentage change in other parameters from baseline to week 24, by arm

Most AEs (Table 3) occurred during the first 24 weeks after switching to B/F/TAF both in the immediate switch arm (study period baseline to week-24) and in the delayed switch arm (study period week-24 to week-48), with the most frequent being headaches, weight gain and central nervous system-related AEs. Figure 2a summarises the percentage change in lipid parameters from baseline to week-24 in both arms showing an improvement in fasting lipids after switching to B/F/TAF. Although there was a higher percentage increase in HDL cholesterol in the delayed switch arm than in the immediate switch arm, the percentage decrease in total cholesterol-to- HDL ratio was higher with B/F/TAF than bPI (-3.1% vs. -1.2%).

Other safety laboratory and clinical parameters were also assessed (Fig. 2b). Renal function assessed using the estimated glomerular filtration rate (eGFR) showed a slight percentage improvement on B/F/TAF with a decrease amongst participants remaining on bPI. At 24 weeks, metabolic parameters assessed were more favourable in those continuing bPI with HbA1c, BMI and weight having a higher percentage increase in those switching to B/F/TAF. The median weight gain in the immediate switch arm was 2.5 kg, (IQR − 0.3 to 3.5 kg).

## Discussion

In this randomised, open label study, we demonstrate that switching virologically suppressive bPI-based ART to B/F/TAF in individuals with historical NRTI resistance maintained virological suppression over 48 weeks. The activity of B/F/TAF in the presence of the 3TC/FTC mutation M184V/I has been confirmed by previous studies, leading to a recent update to the licensed indications [15]. Our study extends the previous data to indicate preserved activity with a history of M184V/I as well as other common NRTI RAMs. These included TAMs known to have the greatest impact on tenofovir susceptibility (M41L, L210W, T215Y) and the tenofovir-associated NAMs K65R and K70E/Q [16–19]. When summing the historical with the baseline resistance data, there were 9 participants with $\:\ge\:$2 high impact TAMs; in addition, 6 participants had tenofovir-associated NAMs. All of these participants maintained virological suppression. Importantly, nearly all (14 of 15) participants with these resistance patterns also had a record of M184V/I, a mutation that reduces the resistance effects of TAMs, K65R and K70E/Q on tenofovir [20–22]. Furthermore, although TAF and TDF have the same resistance profile, in vitro studies suggest that TAF can exert more activity against TDF-resistant viruses with K65R or multiple TAMs, possibly because of high intracellular concentration [16]. These data are consistent with the excellent virological responses we observed after switching bPI-based ART to B/F/TAF, further strengthening the evidence that B/F/TAF retains activity despite the presence of relatively limited NRTI resistance [23].

We used the resistance data obtained at baseline as complementary to rather than as a replacement for the historical resistance data. We recognise that there are limitations with this approach. Previous studies in virologically suppressed individuals generally reported fewer RAMs in HIV-1 DNA compared with historic plasma HIV-1 RNA, which is consistent with our findings [24]. However, the absence of historical RAMs in HIV-1 DNA may reflect both technical and biological factors. Whereas some RAMs may not be archived, others may be archived but escape detection when sampling just a small number of circulating cells. Thus, the loss may only be apparent. However, archived RAMs may also truly disappear over time if the cells that harbour them within transcriptionally active provirus are targeted by an effective immune response. As we lacked the technical ability to discriminate between true and apparent loss, we elected to consider all detected NRTIs RAMs to estimate potential resistance levels [24]. Furthermore, the HIV-1 DNA sequencing data were reported as per the laboratory routine diagnostic protocol applying a conservative frequency cut-off of 15% for reporting RAMs. This made the NGS methodology employed in this study similar to conventional Sanger sequencing in sensitivity. This approach limits the comparison of historical and baseline resistance data as RAMs present at a frequency below 15% in the sample may have been missed.

There were no concerns related to safety and tolerability. More drug-related AEs were observed whilst participants were on B/F/TAF, with a few participants discontinuing the study as a result. Other open label studies have described higher rates of AEs in the switch arm when discontinuing a stable, well-tolerated regimen [4, 25, 26]. Although the sample size was small, we observed a few more AE-related discontinuations than observed in another open label B/F/TAF switch study [4]. In our study, frequently reported AEs on B/F/TAF were headaches, mood disorders (low mood and anxiety), sleep disorders (abnormal dreams, insomnia, and sleep disturbance) and weight gain. These AEs were more common during the first 24 weeks on B/F/TAF and did not appear to occur after this period except for weight gain. Headache was also the most frequently reported AE in another B/F/TAF switch study [4] and was common in naïve B/F/TAF studies [27, 28]. Mood and sleep disorders were common in our study, but rare in other B/F/TAF switch studies [4, 9]; however, insomnia was common in one blinded B/F/TAF naïve study [28]. Consistent with available evidence, we observed more weight gain in participants on B/F/TAF. In a metanalysis that included 8 randomised trials of individuals initiating ART, participants on dolutegravir- and bictegravir-based regimen and those receiving TAF experienced the most weight gain [29]. Current evidence suggests that the weight gain observed when an INSTI is prescribed together with TAF is higher than when either agent is prescribed separately [30, 31]. Nonetheless, just over half of participants in our study discontinued a TDF-based regimen to start B/F/TAF, thus removing the relative weight suppressive effect of TDF [32–35]. Other data suggest a weight-inhibiting effect of TDF that is eliminated when participants switched off TDF [36].

After a switch to B/F/TAF, lipid parameters improved, with decreases in total cholesterol, non-HDL cholesterol and triglycerides. Although the delayed switch arm had higher HDL cholesterol, the ratio of total cholesterol to HDL was lower for B/F/TAF. Other studies have shown that participants on a INSTI containing regimen, including B/F/TAF have lower incidence of dyslipidaemia compared to being on a bPI [37, 38]. The improvement in lipid profile could contribute to reducing cardiovascular disease risk in those on INSTI containing regimen [39].

There was no decrease in estimated glomerular filtration rate on B/F/TAF and no cases of proximal tubulopathy were reported. This was expected based on the reported renal advantages of TAF- over TDF-based regimens [4, 9, 40–42]. An increase in HbA1c from baseline was observed in PIBIK. In Switch studies of B/F/TAF, frequency of hyperglycaemia was higher in the B/F/TAF than comparator regimen [4, 9]. The participants in these switch studies were much younger than the aging HIV cohort which has an average age of greater than 50 years [4, 9]. A large observational study found an increased risk of diabetes/hyperglycaemia when comparing individuals who initiated an INSTI-based vs. non-INSTI-based regimens [43]. In the ADVANCE study, a regimen containing TAF and DTG was associated with an increased risk of type 2 diabetes using a predictive tool that is not validated for people with HIV and black African population [44]. Well conducted large observational studies in real life cohorts with long-term follow up will be required to robustly investigate whether and which INSTI and combination ART are associated with hyperglycaemia.

There are limitations to this study. Recruitment to the study was negatively impacted by the COVID-19 pandemic resulting in recruitment of 72 of the original intended sample size of 100 patients. A few patients in the delayed switch arm experienced delay in switching to B/F/TAF. However, we were able to implement procedures that allowed safe monitoring of patients without compromising the quality of the study.

In summary, the PIBIK study demonstrated that efficacy was maintained in the presence of relatively limited NRTI resistance other than M184V/I when individuals suppressed on a bPI regimen were switched to B/F/TAF. The regimen was safe and generally well tolerated in this small study. Carefully assessing the efficacy of B/F/TAF in individuals with more extensive NRTI resistance is warranted.

## Data Availability

The data that support the findings of this study are not openly available due to reasons of sensitivity and are available from the corresponding author upon reasonable request.

## Abbreviations

RAMS: Resistance Associated Mutations
bPI: Boosted Protease Inhibitor
B/F/TAF: Bictegravir/Emtricitabine/Tenofovir alafenamide
TAMS: Thymidine Analogue Mutations
NRTI: Nucleoside Reverse Transcriptase Inhibitors
NAMS: Nucleoside Analogue Mutations
INSTI: Integrase Strand Transfer Inhibitors
3TC: Lamivudine
FTC: Emtricitabine
ART: Antiretroviral Therapy
PVR: Pure Virologic Response
TDF: Tenofovir Disoproxil Fumarate
DS: Delayed Switch
IS: Immediate Switch
eGFR: Estimated Glomerular Filtration Rate
BMI: Body Mass Index
HDL: High Density Lipoprotein
LDL: Low Density Lipoprotein
TG: Triglycerides
TC: Total Cholesterol
